# Quality of Life in Cohabitants of Patients with Hidradenitis Suppurativa: A Cross-sectional Study

**DOI:** 10.3390/ijerph17166000

**Published:** 2020-08-18

**Authors:** Carmen Ramos-Alejos-Pita, Salvador Arias-Santiago, Alejandro Molina-Leyva

**Affiliations:** 1 Department of Dermatology, Faculty of Medicine, University of Granada, 18016 Granada, Spain; cramale@correo.ugr.es; 2 Department of Dermatology, Hospital Universitario Virgen de las Nieves, 18012 Granada, Spain; alejandro.molina.sspa@juntadeandalucia.es

**Keywords:** hidradenitis suppurativa, quality of life, cohabitants, secondary impact

## Abstract

Hidradenitis suppurativa (HS) is a chronic inflammatory disease that impairs patients’ physical and mental health. However, few studies have considered the consequences of HS on cohabitants. The aims of this study were to explore the impact of HS on the quality of life (QOL) of cohabitants and to assess potentially associated factors. A cross-sectional study was conducted and patients with HS and their cohabitants were invited to participate. Validated questionnaires were used to measure QOL, anxiety and depression, type D personality and sexual dysfunction. The clinical variables of patients and the demographic characteristics of cohabitants were also collected. Twenty-seven patients and 27 cohabitants were included for analysis. Patients and cohabitants presented significant QOL impairment. A direct association was found between the Dermatology Life Quality Index (DLQI) and the Familiar Dermatology Life Quality Index (FDLQI). DLQI scores were associated with the presence of negative affectivity, a trait typical of type D personality, as well as with cohabitants’ anxiety. FDLQI scores were associated with cohabitant anxiety and patient depression. Hidradenitis suppurativa damages quality of life in patients and cohabitants. Identifying potential psychological factors could help us to recognize at-risk patients and apply personalized treatments for them and their environment.

## 1. Introduction

Hidradenitis suppurativa (HS) is a genetically heterogeneous chronic inflammatory skin disease with a reported prevalence of about 1%. It is characterized by nodules, abscesses, fistulae and large scarred areas and usually accompanied by pain and purulent secretions with unpleasant odor [1,2]. Obesity, tobacco and hormonal disorders have been postulated as the main factors that influence the onset and evolution of the disease [2], and it has been associated with multiple physical comorbidities such as spondyloarthritis, inflammatory bowel disease and increased cardiovascular risk [3,4]. Since HS is an autoinflammatory skin disorder [5,6] with a great clinical heterogeneity, a growing body of comorbidities both cutaneous [7] and extracutaneous [3,4,8] were claimed to be linked to it. Despite the epidemiological association, the exact pathogenetical mechanism is still elusive and may be of pivotal importance in improving patients and relative’s quality of life.

Furthermore, HS impairs patients’ mental health. It has been related to higher levels of depression and anxiety, worse quality of life, sexual dysfunction and a higher suicide risk [3,4,9,10,11] Moreover, it has direct consequences on social relationships and professional careers, such as higher absenteeism and unemployment levels [3,4]. For all these reasons, HS is a stigmatizing and disabling disease.

However, few studies have considered the consequences of HS on the people who live with patients. We argue that this disease could have a negative effect on cohabitants’ mental health because HS requires continuous skin care and can have an impact on the household economy.

The aims of this study are to analyze quality of life in cohabitants of people with hidradenitis suppurativa, to assess the potential related factors and to explore the impact that the disease may have on them. These objectives could help us to propose new multidisciplinary approaches, which include patients and their close environment.

## 2. Materials and Methods

### 2.1. Design and Study Population

We conducted a cross-sectional study that included patients with HS and their cohabitants. Participants were recruited consecutively from 1 January to 31 March 2020 from patients who attended a monographic consultation for HS in the reference area of the Hospital Universitario Virgen de las Nieves (Granada, Spain). The study was approved by the Provincial Research Ethics Committee of Granada. The ethical code is 0105-N-20. Participants received all the information and gave their informed written consent prior to completing the questionnaires. In addition, all the data was processed in accordance with current legislation to preserve the autonomy and privacy of patients and cohabitants.

**Figure 1:**
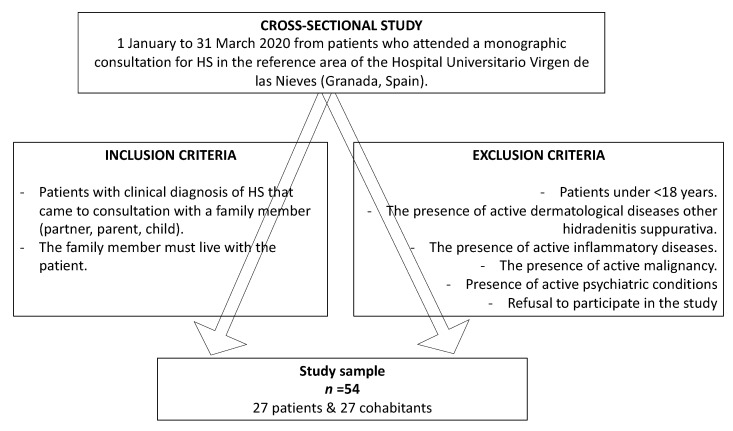
Flowchart. Inclusion and exclusion criteria.

Patients and cohabitants who came to the consultation together were invited to participate. Inclusion criteria were as follows: patients diagnosed with HS at any stage of severity and their family members (partner, parent, child) who also live with the patient. Exclusion criteria were as follows: <18 years, the presence of several physical or psychological illnesses and refusal to participate in the study. Participants who met the inclusion and exclusion criteria were given a questionnaire which they completed after the consultation. Figure 1.

### 2.2. Study Variables

Quality of life. The Dermatology Life Quality Index (DLQI) [12,13] and the Family Dermatology Life Quality Index (FDLQI) [14,15] questionnaires were used to measure quality of life in patients and cohabitants respectively. Both questionnaires are validated on the Spanish population. They have 10 questions, which are scored from 0 to 3 on a Likert scale. The overall score ranges from 0 to 30, using the following categories: no impact (0–1), mild (2–5), moderate (6–10), severe (11–20) and very severe. A higher DLQI score correlates with worse quality of life (21–30).

Anxiety and Depression. The Hospital Anxiety and Depression Scale (HADS) [16], validated on the Spanish population, was used to evaluate the prevalence of anxiety and depression. This questionnaire consists of 14 items, each one scored using an adapted Likert scale. It is divided into two scales of seven items each. Scores ≥8 on the subscales were considered indicative of anxiety or depression.

Type D personality. Type D personality was assessed using the DS14 questionnaire [17], validated on the Spanish population. It has 14 items, 7 for negative affectivity and 7 for social inhibition. Scores ≥10 in both subscales were indicative of type D personality.

Sexual dysfunction. The International Index of Erectile Function, IIEF-5 [18] and the Female Sexual Function Index, FSFI-6 [19] questionnaires were used to collect the prevalence of sexual dysfunction in men and women, respectively. Both questionnaires are validated on the Spanish population. Among cohabitants, it was given only to those who were the patient’s partner. Scores ≤21 were considered significant for IIEF-5. Scores ≤19 were considered significant for FSFI-6. In addition, questions were added about sex life: a numerical scale to express their degree of subjective affect and questions about the main factors that impair their sex life.

HS severity. The severity of HS was determined by the Hurley stage [20] and the International Hidradenitis Suppurativa Severity Score System (IHS4) [21], which were collected at the time of consultation. Hurley Stadium has 3 stages: I (abscesses, single or multiple, without fistulae or scars), II (recurrent abscesses, fistulae and scars, single or multiple, widely separated from each other) and III (abscesses and fistulae with large areas of extensive scarring). The IHS4 is a more specific classification for the degree of inflammation. Its score is calculated as follows: (nº inflammatory nodules × 1) + (nº abscesses × 2) + (nº fistulae × 4), with the following cut-off points: mild (<4), moderate (4–10) and severe (>10).

Other variables of interest. Demographic data was obtained for both groups (age, sex, marital status, educational level and occupation) and clinical data on patients (age at onset and current treatment). Patients were treated according to current European HS guidelines [20]. In addition, the relationship with the patient was collected for all cohabitants.

### 2.3. Data Analysis

Continuous variables were expressed as the mean and standard deviation (SD) and the Wilcoxon–Mann–Whitney test was used for comparison. Qualitative variables were expressed as proportions and compared using the Chi-square test or Fisher’s exact test, where necessary. In these cases, *p* < 0.05 was considered statistically significant. Simple linear regression was used to analyze the relationship between DLQI/FDLQI and continuous variables, and analysis of variance ANOVA was used when the variables were qualitative. To control the error derived from multiple comparisons, Bonferroni correction was applied and *p* < 0.0021 (0.05/24) was considered statistically significant. The data was analyzed using JMP 14.1 (SAS Institute, Cary, NC, USA).

## 3. Results

A total of 54 participants, 27 patients and 27 cohabitants, were included. Both groups showed similar socio-demographic characteristics, which are summarized in supplemental material Table S1. There was a higher proportion of women in both groups and, in relation to marital status, a higher proportion of married/partner. Among patients, the most frequent Hurley’s stage was II and the mean value for the IHS4 was moderate. The patients’ clinical characteristics are shown in supplemental material Table S2.

DLQI scores showed the disease’s severe impact on patients’ quality of life, while cohabitants obtained moderate values for FDLQI. Scores for negative affectivity and depression were significantly higher in patients. Anxiety levels and sexual dysfunction were higher in patients compared to cohabitants, with a tendency to statistical significance. Table 1 shows the results from the patients’ and cohabitants’ questionnaires.

The univariate analysis of the patients’ quality of life (Table 2) showed a significant association between DLQI and three variables: FDLQI, patients’ negative affectivity and cohabitants’ anxiety. In addition, a trend to significance was observed between DLQI and IHS4, as well as with patients’ anxiety and depression.

Table 3 shows the univariate analysis of the cohabitants’ quality of life. They showed a direct association between FDLQI and DLQI, cohabitants’ anxiety and patients’ depression. A trend to significance was found between FDLQI and the following variables: IHS4, patients’ anxiety, negative affectivity or sexual dysfunction and cohabitants’ sexual dysfunction. In addition, the analyses showed an inverse association between BMI of the patients and cohabitants’ quality of life, with a trend to significance.

**Figure 2:**
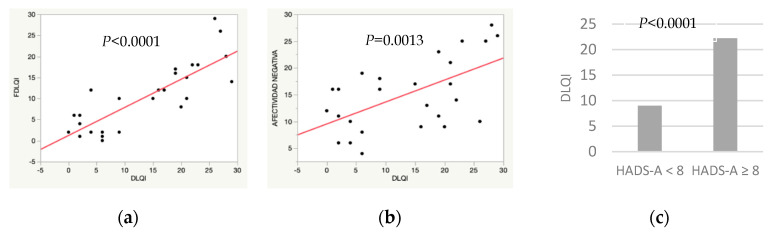
Potential factors associated with worse quality of life in patients with hidradenitis suppurativa. (**a**) Simple linear regression. Familiar Dermatology Life Quality Index (FDLQI)/Dermatology Life Quality Index (DLQI). (**b**) Simple linear regression. Negative affectivity, DS14 test/DLQI. (**c**) χ2 test. Cohabitants’ anxiety (Hospital Anxiety and Depression Scale, anxiety subscale (HADS-A) ≥8)/DLQI.

**Figure 3:**
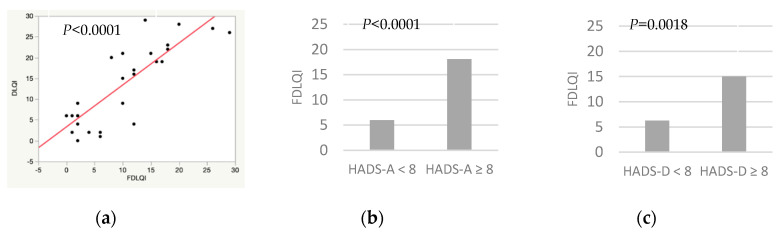
Potential factors associated with worse quality of life in cohabitants of patients with hidradenitis suppurativa. (**a**) Simple linear regression. Dermatology Life Quality Index (DLQI)/Familiar Dermatology Life Quality Index (FDLQI). (**b**) χ2 test. Cohabitants’ anxiety (Hospital Anxiety and Depression Scale, anxiety subscale (HADS-A) ≥8)/FDLQI. (**c)** χ2 test. Patients’ depression (Hospital Anxiety and Depression Scale, depression subscale (HADS-D) ≥8)/FDLQI.

Figure 2 and Figure 3 show the correlation between DLQI/FDLQI and potential factors affecting quality of life in patients and cohabitants, respectively. Figure 2a and Figure 3a show a direct association between DLQI and FDLQI. Figure 2b show that a higher DLQI score is associated with a higher negative affectivity and anxiety in cohabitants. Depression in patients, Figure 2c, and anxiety in cohabitants, Figure 3b,c, are also associated with a higher FDLQI.

## 4. Discussion

The impact of hidradenitis suppurativa on patients’ quality of life has been widely studied. Furthermore, HS has been shown to induce more damage than other skin diseases [22]. However, few studies have addressed the effect of HS on cohabitants’ quality of life [23,24].

Wlodarek et al. [23] were the first to report that quality of life was diminished in family members. This study only included patients’ partners, whether they were cohabitants or not, and they found a direct relationship between the impairment and the disease severity. Subsequently, Marasca et al. [24] linked patients’ quality of life to that of cohabitants, showing that both were affected. They also reported that the impact was higher in women and when the cohabitant was the patient’s partner. Our study is the first to include psychological variables that can impair quality of life, such as the presence of anxiety, depression, sexual dysfunction and type D personality.

We found that quality of life was diminished in cohabitants. The FDLQI reflects a moderate impact which is similar to previous studies (8.7 ± 6.8 in Wlodarek et al. and 10.11 in Marasca et al.). These results can be explained because in all three studies, the proportion of patients categorized as Hurley II was predominant and similar (51.85% vs. 60% vs. 45.8%, respectively).

Previous studies showed a significant association between cohabitants’ quality of life and disease severity, which is in line with the trend of significance found in our analysis. This suggests that the severity of HS could play a significant role in the quality of life impairment in patients and partners, not only because of skin symptoms, but also because of continuous skin care, more uncomfortable treatments and greater economic expenditure.

Marasca et al. reported differences for some sociodemographic variables: women obtained higher DLQI scores compared to men, FDLQI scores were lower for cohabitants with higher education levels and patients’ partners reported higher FDLQI scores than other family members. We found a possible association between sexual dysfunction in couples and poorer quality of life in cohabitants. Our results may be in line with the higher FDLQI score of couples described by Marasca et al. This highlights the importance that the sexual sphere can have on people’s well-being and we should consider it in the integral evaluation of the patient. However, in line with Wlodarek et al., our study found no significant differences between men’s and women’s quality of life. We consider that, despite our small sample, the strong relationship between factors allowed us to obtain clinically and statistically significant results, which were not found for the sex variable. This, in conjunction with the results of Wlodarek et al., makes us think that the gender differences reflected by Marasca et al. may not be as relevant.

Additionally, in agreement with previous studies, we observed a strong relationship between patients’ and cohabitants’ quality of life. We have also evaluated psychological and personality factors potentially related to disease adaptation and consequently to quality of life. Cohabitant anxiety was associated with a diminished quality of life in both groups. Depression in patients was associated with a worse quality of life in their cohabitants. Patients with a higher score for negative affectivity showed poorer quality of life. This makes us think that anxiety, depression and type D personality could have a fundamental role in the mental well-being of patients and their cohabitants. Furthermore, early detection and appropriate treatment could have a positive impact on the natural history and personal experience of the disease.

Other factors studied showed a potential relationship with patients’ and cohabitants’ quality of life and, although not statistically significant, they could become clinically relevant. Our study suggests that patients’ quality of life may be affected by the severity of the disease and by patient anxiety and depression. On the other hand, a poorer quality of life in cohabitants could be conditioned by the severity of the disease, patient anxiety, depression or negative affectivity and the prevalence of sexual dysfunction. These results suggest that further research is needed to increase our knowledge of the factors that influence quality of life in patients and cohabitants.

These results reaffirm that HS has a negative impact on the quality of life and mental health of patients and also impairs the well-being of the people living with them.

The limitations of this study were: (1) The small sample size. However, the strong association between variables has shown clinically and statistically significant results. (2) The inclusion of participants was conditioned by the fact that patients and cohabitants came to the consultation together. This excluded unaccompanied patients from the study, which could have led to differential selection bias. (3) The cross-sectional design of the study influenced patient responses, according to the disease control at the time. For all these reasons, we believe that new studies with a larger sample size are needed in order to study other potential factors associated with poorer quality of life in patients and cohabitants.

## 5. Conclusions

Hidradenitis suppurativa impairs quality of life in patients and cohabitants. We have identified psychological factors potentially related to a worse quality of life such as anxiety, depression, negative affectivity or the quality of life of the people they live with. Taking into account these factors could help us identify at-risk patients in order to apply a personalized and needs-oriented approach to patients and their cohabitants. It is necessary to underline the importance of addressing hidradenitis suppurativa from an integral point of view, which goes beyond cutaneous manifestations and takes into account the psychosocial aspects of the disease.

## Figures and Tables

**Table 1:** Patients and cohabitants’ questionnaires results.

Variables	Patients (*n* = 27)	Cohabitants (*n* = 27)	*p*
DLQI	13.88 (SD 9.53)	-	-
FDLQI	-	10.48 (SD 7.76)	-
Type D Personality Negative Affectivity Score (NA) Social Inhibition Score (SI) NA + SI ≥ 10	15.22 (SD 6.68) 10.85 (SD 5.40) 51.85% (14/27)	11.29 (SD 6.99) 10.11 (SD 7.21) 44.44% (12/27)	**0.03** 0.67 0.58
Anxiety HADS Score HADS-A ≥ 8	9.51 (SD 4.89) 62.96% (17/27)	7.22 (SD 4.20) 37.03% (10/27)	0.07 0.05
Depression HADS Score HADS-D ≥ 8	7.70 (SD 5.11) 48.14% (13/27)	5.14 (SD 4.52) 18.51% (5/27)	0.05 **0.02**
Sexual Dysfunction IIEF-5/FSFI-6 Score IIEF-5 ≤ 21/FSFI-6 ≤ 19	18.26 (SD 6.28) 50.00% (13/26)	20.82 (SD 6.51) 35.29% (6/17)	0.20 0.34

DLQI, Dermatology Life Quality Index; FDLQI, Familiar Dermatology Life Quality Index; DS14, Type D personality scale; NA, Negative affectivity; SI, Social inhibition; HADS, Hospital Anxiety and Depression Scale; IIEF-5, International Index of Erectile Function; FSFI-6, Female Sexual Function Index. Continuous variables are expressed as mean and standard deviation (SD). Qualitative variables are expressed as proportions. Wilcoxon–Mann–Whitney test was used for comparison between continuous variables. To compare qualitative variables, Chi-square test or Fisher’s exact test when necessary was used.

**Table 2:** Univariate analysis of potentially factors associated with quality of life in HS patients.

Variables	DLQI	*p*
Age, Years	−0.02 (SD 0.13)	0.85
Sex Female Male	15.40 (SD 2.46) 12.00 (SD 2.76)	0.36
Marital status Single Partner Married Divorced Widowed	9.33 (SD 5.61) 13.00 (SD 3.43) 15.18 (SD 2.43)	0.60
Educational Level None Primary or Equivalent Secondary or Equivalent Vocational Training University or Higher	6.00 (SD 9.34) 11.27 (SD 2.81) 13.25 (SD 4.67) 13.20 (SD 4.17) 21.00 (SD 3.81)	0.30
Occupation Employee Public Worker Freelancer Retiree Unemployed Student Other	17.16 (SD 4.01) 8.00 (SD 4.91) 19.00 (SD 9.82) 9.25 (SD 4.91) 16.62 (SD 3.47) 19.00 (SD 9.82) 10.66 (SD 5.67)	0.61
Patients’ BMI	−0.09 (SD 0.23)	0.67
Hurley I II III	11.50 (SD 3.31) 13.00 (SD 2.50) 20.20 (SD 4.19)	0.25
IHS4	0.56 (SD 0.20)	0.01 *
Treatment Topical Systemic Drugs (+/- topical) Biological Drugs (+/- topical) Biological Drugs + Systemic (+/- topical) Surgery (+/- topical) Surgery + Systemic Drugs (+/- topical)	2.00 (SD 9.63) 12.25 (SD 2.78) 17.33 (SD 5.56) 18.80 (DS 4.30) 16.00 (SD 4.81) 8.00 (SD 6.80)	0.49
Cohabitants’ FDLQI	1.00 (SD 0.14)	**<0.0001**
Negative Affectivity (NA)	0.83 (SD 0.23)	**0.0013**
Social Inhibition (SI)	0.38 (SD 0.34)	0.27
Type D personality NA + SI < 10 NA + SI ≥ 10	12.30 (SD 2.66) 15.35 (SD 2.56)	0.41
Cohabitants’ Negative Affectivity	0.21 (SD 0.26)	0.42
Cohabitants’ Social Inhibition	0.38 (SD 0.25)	0.14
Cohabitants’ Type D Personality	3.20 (SD 3.71)	0.39
Anxiety HADS-A < 8 HADS-A ≥ 8	7.90 (SD 2.67) 17.41 (SD 2.05)	0.0093 *
Cohabitants’ Anxiety HADS-A < 8 HADS-A ≥ 8	9.00 (SD 1.72) 22.20 (SD 2.25)	**<0.0001**
Depression HADS-D < 8 HADS-D ≥ 8	8.78 (SD 2.14) 19.38 (SD 2.22)	0.0021 *
Cohabitants’ Depression HADS-D < 8 HADS-D ≥ 8	12.63 (SD 1.99) 19.40 (SD 4.17)	0.15
Sexual Dysfunction IIEF-5 > 21/FSFI-6 > 19 IIEF-5 ≤ 21/FSFI-6 ≤ 19	12.00 (SD 2.57) 16.69 (SD 2.57)	0.21
Partners’ Sexual Dysfunction IIEF-5 > 21/FSFI-6 > 19 IIEF-5 ≤ 21/FSFI-6 ≤ 19	11.18 (SD 2.59) 18.33 (SD 3.51)	0.12
Relationship Patient - Cohabitant Partner Father/Mother–Son/Daughter Son/Daughter–Father/Mother	15.30 (SD 2.64) 17.50 (SD 6.73) 9.25 (SD 4.76)	0.48
Years of Evolution	−0.13 (SD 0.29)	0.66

DLQI, Dermatology Life Quality Index; BMI, Body mass index; IHS4, International Hidradenitis Suppurativa Severity Score System; FDLQI, Familiar Dermatology Life Quality Index; DS14, Type D personality scale; NA, Negative affectivity; SI, Social inhibition; HADS, Hospital Anxiety and Depression Scale; IIEF-5, International Index of Erectile Function; FSFI-6, Female Sexual Function Index. Simple linear regression was used for comparisons between DLQI and continuous variables: data are expressed as beta coefficient (standard deviation). For comparisons between DLQI and qualitative variables, the analysis of variance ANOVA was used: data are expressed as mean (standard deviation). *p* < 0.0021 were considered statistically significant and are highlighted in bold. *p* with a tendency for significance are highlighted with *.

**Table 3:** Univariate analysis of potentially factors associated with quality of life in cohabitants.

Variables	FDLQI	*p*
Age, Years	0.08 (SD 0.10)	0.44
Sex Female Male	9.29 (SD 1.87) 12.50 (SD 2.45)	0.30
Marital Status Single Partner Married Divorced Widowed	8.00 (SD 4.57) 8.85 (SD 2.99) 10.64 (SD 2.11) 10.00 (SD 7.93) 19.00 (SD 5.60)	0.58
Educational Level None Primary or Equivalent Secondary or Equivalent Vocational Training University or Higher	9.00 (SD 5.75) 13.00 (SD 2.87) 10.80 (DS 3.64) 7.71 (SD 3.07) 10.60 (SD 3.64)	0.79
Occupation Employee Public Worker Freelancer Retiree Unemployed Student Other	18.25 (SD 3.51) 6.00 (SD 3.14) 15.00 (SD 4.97) 13.16 (SD 2.87) 7.16 (SD 2.87) 10.00 (SD 4.97) 4.00 (SD 4.97)	0.12
Patients’ BMI	−0.45 (SD 0.23)	0.06 *
Hurley I II III	8.87 (SD 2.65) 9.28 (SD 2.00) 16.40 (SD 3.35)	0.16
IHS4	0.34 (SD 0.18)	0.06 *
Treatment Topical Systemic Drugs (+/− topical) Biological Drugs (+/− topical) biological Drugs + Systemic (+/− topical) Surgery (+/− topical) Surgery + Systemic Drugs (+/- topical)	4.00 (SD 8.30) 10.91 (SD 2.39) 11.66 (SD 4.79) 13.00 (SD 3.71) 8.00 (SD 4.15) 8.00 (SD 5.87)	0.88
Patients’ DLQI	0.66 (SD 0.09)	**<0.0001**
Negative Affectivity (NA)	0.05 (SD 0.22)	0.80
Social Inhibition (SI)	0.17 (SD 0.21)	0.42
Type D Personality NA + SI < 10 NA + SI ≥ 10	9.26 (SD 2.01) 12.00 (SD 2.24)	0.37
Patients’ Negative Affectivity	0.50 (SD 0.21)	0.02 *
Patients’ Social Inhibition	0.18 (SD 0.28)	0.53
Patients’ Type D Personality	0.78 (SD 3.04)	0.79
Anxiety HADS-A < 8 HADS-A ≥ 8	6.00 (SD 1.23) 18.10 (SD 1.60)	**<0.0001**
Patients’ Anxiety HADS-A < 8 HADS-A ≥ 8	7.00 (SD 2.34) 12.52 (SD 1.79)	0.07 *
Depression HADS-D < 8 HADS-D ≥ 8	9.72 (SD 1.65) 13.80 (SD 3.46)	0.29
Patients’ Depression HADS-D < 8 HADS-D ≥ 8	6.28 (SD 1.73) 15.00 (SD 1.80)	**0.0018**
Sexual Dysfunction IIEF-5 > 21/FSFI-6 > 19 IIEF-5 ≤ 21/FSFI-6 ≤ 19	8.81 (SD 2.26) 15.33 (SD 3.07)	0.10 *
Partners’ Sexual Dysfunction IIEF-5 > 21/FSFI-6 > 19 IIEF-5 ≤ 21/FSFI-6 ≤ 19	8.4 (SD 2.35) 15.00 (SD 2.81)	0.09 *
Relationship Patient - Cohabitant Partner Father/Mother–Son/Daughter Son/Daughter–Father/Mother	10.55 (SD 1.88) 8.00 (SD 4.62) 11.50 (SD 3.27)	0.82
Years of Evolution	0.05 (SD 0.08)	0.48

FDLQI, Familiar Dermatology Life Quality Index; BMI, Body mass index; IHS4, International Hidradenitis Suppurativa Severity Score System; FDLQI, Familiar Dermatology Life Quality Index; DS14, Type D personality scale; NA, Negative affectivity; SI, Social inhibition; HADS, Hospital Anxiety and Depression Scale; IIEF-5, International Index of Erectile Function; FSFI-6, Female Sexual Function Index. Simple linear regression was used for comparisons between DLQI and continuous variables: data are expressed as beta coefficient (standard deviation). For comparisons between DLQI and qualitative variables, the analysis of variance ANOVA was used: data are expressed as mean (standard deviation). *p* < 0.0021 were considered statistically significant and are highlighted in bold. *p* with a tendency for significance are highlighted with *.

## Supplementary Materials

The following are available online at https://www.mdpi.com/1660-4601/17/16/6000/s1, Table S1: Sociodemographic variables of patients and cohabitants; Table S2: Clinical variables of patients with hidradenitis suppurativa.
