# Supplementary material for: Quality of Life in Cohabitants of Patients with Hidradenitis Suppurativa: A Cross-sectional Study
Source: Int J Environ Res Public Health. 2020 Aug 18;17(16):6000. doi: 10.3390/ijerph17166000 (PMC7459451; doi:10.3390/ijerph17166000)
Supplement: Supplementary file 1 [file ijerph-17-06000-s001.pdf]

Table 1. sup. Sociodemographic variables of patients and cohabitants.

Variables	Patients (n=27)	Cohabitants (n=27)	*p*
Age, Years	41.81 (SD 14.54)	45.18 (SD 14.34)	0.39
Sex (female:male)	15:12	17:10	0.57
Marital Status			
Single	11.11% (3/27)	11.11% (3/27)	0.52
Partner	29.63% (8/27)	25.92% (7/27)	
Married	59.25% (16/27)	51.85% (14/27)	
Divorced	0.00% (0/27)	3.70% (1/27)	
Widows	0.00% (0/27)	7.40% (2/27)	
Educational Level			
None	3.70% (1/27)	7.40% (2/27)	0.85
Primary or Equivalent	40.74% (11/27)	29.63% (8/27)	
Secondary or Equivalent	14.81% (4/27)	18.51% (5/27)	
Vocational Training	18.51% (5/27)	25.92% (7/27)	
University or Higher	22.22% (6/27)	18.51% (5/27)	
Occupation			
Employee	22.22% (6/27)	14.81% (4/27)	0.91
Public Worker	14.81% (4/27)	18.51% (5/27)	
Freelancer	3.70% (1/27)	7.40% (2/27)	
Retiree	14.81% (4/27)	22.22% (6/27)	
Unemployed	29.63% (8/27)	22.22% (6/27)	
Student	3.70% (1/27)	7.40% (2/27)	
Other	11.11% (3/27)	7.40% (2/27)	
Relationship with the Patient			
Partner	-	66.66% (18/27)	-
Son/Daughter		11.11% (3/27)	
Father/Mother		22.22% (6/27)	

Continuous variables are expressed as mean and standard deviation (SD). Qualitative variables are expressed as proportions. Wilcoxon-Mann-Whitney test was used for comparison between continuous variables. To compare qualitative variables, Chi-square test or Fisher's exact test when necessary was used.

Table 2. sup. Clinical variables of patients with hidradenitis suppurativa.

Variables	Patients (n=27)
BMI	30.36 (SD 6.16)
Hurley	
I	29.63% (8/27)
II	51.85% (14/27)
III	18.51% (5/27)
IHS4	8.14 (SD 8.03)
Age of onset (years)	27.40 (SD 13.51)
Years of evolution	14.40 (SD 14.17)
Treatment	
Topical	3.70% (1/27)
Systemic Drugs (+/- topical)	44.44% (12/27)
Biological Drugs (+/- topical)	11.11% (3/27)
Systemic + Biological Drugs (+/- topical)	18.51% (5/27)
Surgery (+/- topical)	14.81% (4/27)
Surgery + Systemic Drugs (+/- topical)	7.40% (2/27)

BMI, body mass index; IHS4, International Hidradenitis Suppurativa Severity Score System. Continuous variables are expressed as mean and standard deviation (SD). Nominal variables are expressed as proportions.
